# Effect of temperature and colonization of *Legionella pneumophila* and *Vermamoeba vermiformis* on bacterial community composition of copper drinking water biofilms

**DOI:** 10.1111/1751-7915.12457

**Published:** 2017-01-18

**Authors:** Helen Y. Buse, Pan Ji, Vicente Gomez‐Alvarez, Amy Pruden, Marc A. Edwards, Nicholas J. Ashbolt

**Affiliations:** ^1^ Pegasus Technical Services, Inc c/o US EPA26 W Martin Luther King Drive NG‐16CincinnatiOH45268USA; ^2^ Department of Civil and Environmental EngineeringVirginia TechBlacksburgVAUSA; ^3^ School of Public HealthUniversity of AlbertaEdmontonAB T6G 2G7Canada

## Abstract

It is unclear how the water‐based pathogen, *Legionella pneumophila* (Lp), and associated free‐living amoeba (FLA) hosts change or are changed by the microbial composition of drinking water (DW) biofilm communities. Thus, this study characterized the bacterial community structure over a 7‐month period within mature (> 600‐day‐old) copper DW biofilms in reactors simulating premise plumbing and assessed the impact of temperature and introduction of Lp and its FLA host, *Vermamoeba vermiformis* (Vv), co‐cultures (LpVv). Sequence and quantitative PCR (qPCR) analyses indicated a correlation between LpVv introduction and increases in *Legionella* spp. levels at room temperature (RT), while at 37°C, Lp became the dominant *Legionella* spp. qPCR analysis suggested Vv presence may not be directly associated with Lp biofilm growth at RT and 37°C, but may contribute to or be associated with non‐Lp legionellae persistence at RT. Two‐way PERMANOVA and PCoA revealed that temperature was a major driver of microbiome diversity. Biofilm community composition also changed over the seven‐month period and could be associated with significant shifts in dissolved oxygen, alkalinity and various metals in the influent DW. Hence, temperature, biofilm age, DW quality and transient intrusions/amplification of pathogens and FLA hosts may significantly impact biofilm microbiomes and modulate pathogen levels over extended periods.

## Introduction

Biofilms are an important source of microorganisms within drinking water (DW) systems, where sessile growth confers a growth advantage compared to their planktonic counterparts [reviewed in Berry *et al*., 2006]. Drinking water biofilms are ubiquitous within distribution systems and in premise (i.e. building) plumbing, providing an ideal microenvironment for a diverse array of microorganisms, with their development influenced by water chemistry (Ji *et al*., 2015); disinfectant residual (Shen *et al*., 2016); pipe material/corrosion products (Yu *et al*., 2010; Buse *et al*., 2013b; Lin *et al*., 2013; Buse *et al*., 2014); biofilm age (Martiny *et al*., 2003; Lee *et al*., 2005); and composition of upstream microbiomes (Pinto *et al*., 2012; Lu *et al*., 2014). Drinking water biofilms are viewed as a source of pathogens as they provide a reservoir for their accumulation/growth and subsequent release. Opportunistic, water‐based pathogens, such as representative *Legionella* spp., *Mycobacterium* spp. and *Pseudomonas* spp., have all been reported to grow within DW biofilms (Falkinham *et al*., 2001; Wingender and Flemming, 2004; Feazel *et al*., 2009; Buse *et al*., 2014; Lu *et al*., 2014). In particular, *L. pneumophila* may now account for some 66% of DW‐related outbreaks in the United States, noting a 286% increase in legionellosis cases between 2000 and 2014 (0.42–1.62 cases per 100 000 persons; Garrison *et al*., 2016). Similarly in Europe, between 2012 and 2014, an increase in Legionnaires’ disease cases (5852–6941 respectively) was also observed with the majority of those due to *L. pneumophila* and hot‐ and cold‐water systems and cooling towers being implicated as likely sources (ECDC, 2016). Coupled with the significant healthcare costs due to Legionnaires’ disease and non‐tuberculous mycobacteria (with over 400 and 6000 hospital cases and associated healthcare costs of $9.4 and 46 million between 2004 and 2007 in the United States respectively; Collier *et al*., 2012), these water‐based pathogens are a major concern from economic and public health standpoints.

Drinking water biofilm development has been shown to differ based on plumbing surface material such as chlorinated, plasticized and unplasticized polyvinylchloride (cPVC, pPVC and uPVC, respectively), polybutylene, cast iron, cross‐linked polyethylene (PEX), copper (Cu), stainless steel (SS) and glass (Rogers and Keevil, 1992; Rogers *et al*., 1994; Armon *et al*., 1997; Kuiper *et al*., 2004; Lehtola *et al*., 2007; Moritz *et al*., 2010; Valster *et al*., 2010; Messi *et al*., 2011; Lin *et al*., 2013). Unfortunately, most biofilm‐related studies to date are on the order of days to weeks with water chemistries not representative of DW, and longer‐term comparisons of *L. pneumophila*‐associated biofilm growth between surface materials are both rare and often conflicting. After laboratory inoculation of *L. pneumophila* into biofilm reactors operated at 20°C, culturable *L. pneumophila* can persist for up to 4 weeks in PEX‐ and Cu‐grown DW biofilms and up to 4 months in uPVC‐grown DW biofilms (Moritz *et al*., 2010; Buse *et al*., 2013b). However, at the same temperature, *L. pneumophila* culturability was not supported within Cu DW biofilms compared to growth within polybutylene and cPVC DW biofilms; yet at 40°C, *L. pneumophila* was culturable for up to 3 weeks within Cu DW biofilms in the same study (Rogers *et al*., 1994). Survival of *L. pneumophila* was enhanced on PVC‐grown relative to glass‐grown DW biofilms (Armon *et al*., 1997), while colonization of Cu‐grown DW biofilms occurred more readily and persistently than within uPVC‐grown DW biofilms (Buse *et al*., 2013). Collectively, these studies indicate that legionellae‐related biofilm development is a common, yet complex phenomenon that is heavily impacted by temperature, surface material, water quality and age, among other factors. Thus, it is difficult to interpret key factors impacting *L. pneumophila* biofilm colonization; however, characterizing the broader bacterial composition of biofilms could draw further insight. This could allow comparisons to be made across studies, providing principles of *L. pneumophila* ecological interactions and potentially explaining otherwise aberrant observations regarding the factors influencing its colonization of biofilms.

Free‐living amoebae (FLA), such as *Acanthamoeba* spp. and *Vermamoeba* (formerly *Hartmannella*) *vermiformis*, are also a public health concern as they are known hosts for *L. pneumophila* (Harb *et al*., 2000; Lau and Ashbolt, 2009) and have been co‐isolated from *L. pneumophila*‐contaminated systems (Yamamoto *et al*., 1992; Thomas *et al*., 2006, 2008). *Acanthamoeba* spp. are less abundant in freshwater and DW systems compared to *V. vermiformis* (Valster *et al*., 2010; Buse *et al*., 2013a), with the latter demonstrated to be the prominent host for *L. pneumophila* in DW derived from hospital and recreational building hot‐water tanks (Wadowsky *et al*., 1988; Fields *et al*., 1989), within polyethylene‐ and pPVC‐grown DW biofilms (Kuiper *et al*., 2004; Valster *et al*., 2010) and within SS biofilms composed of *P. aeruginosa*,* Klebsiella pneumoniae* and *Flavobacterium* spp. (Murga *et al*., 2001). A previous study reported high infectivity and intracellular replication of *L. pneumophila* strain Chicago‐2 within the strain CDC‐19 of permissive amoeba host, *V. vermiformis* (Buse and Ashbolt, 2011); thus, both were chosen for use in this study.

The aim of this study was to profile bacterial diversity and relative abundance within complex, mature DW biofilms (initially grown on Cu surfaces at pH > 8.2 for over 600 days) and track taxonomic changes as a function of operating temperature and introduction of *L. pneumophila* and *V. vermiformis* leading to colonization over an extended period (7 months). The overall goal of this work was to advance understanding of complex microbial niches conducive to water‐based pathogen colonization and survival within DW biofilms.

## Results

### Quantification of *Legionella* spp., *L. pneumophila* and *V. vermiformis* within Cu DW biofilms

**Figure 1:**
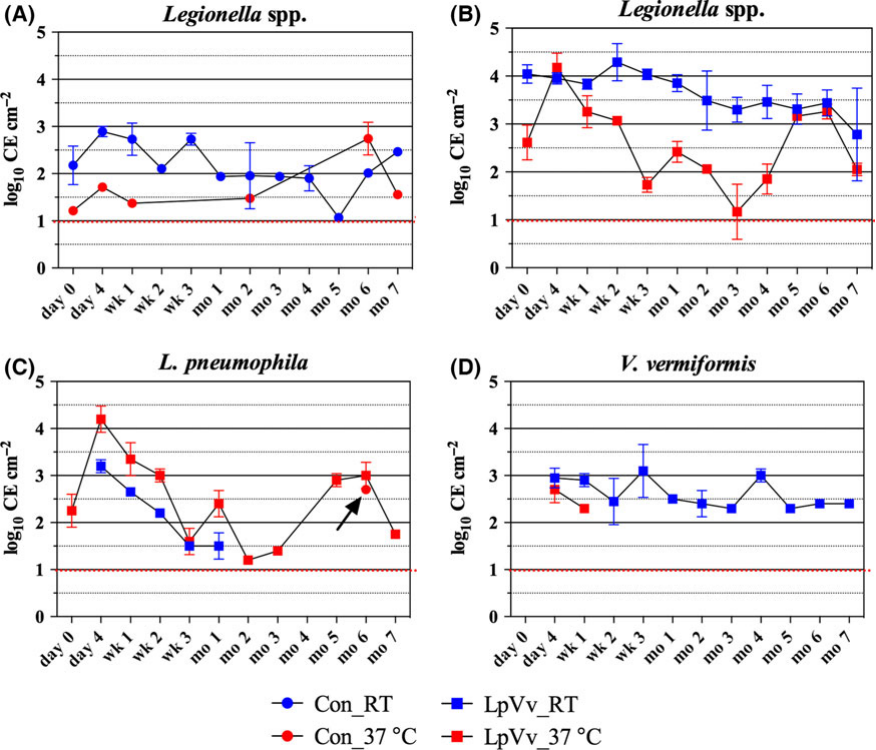
Quantitative PCR (qPCR) analysis of *Legionella* spp., *Legionella pneumophila* and *Vermamoeba vermiformis*. Biofilm samples were subjected to qPCR assays targeting the 16S rRNA gene of *Legionella* spp. (A, B), the *sidF* gene of *L. pneumophila* (C) and the 18S rRNA gene of *V. vermiformis* (D). Data represent the mean for duplicate samples with standard error mean bars. *Legionella* spp. quantification data for control (Con) and inoculated (LpVv) biofilm samples are shown separately (A and B respectively). *Legionella pneumophila* was not detected in the control reactors except at the 6‐month time point (indicated by the black arrow, C). The limit of quantification of 1 log_10_ CE cm^−2^ is indicated by the red dotted line.

**Figure 2:**
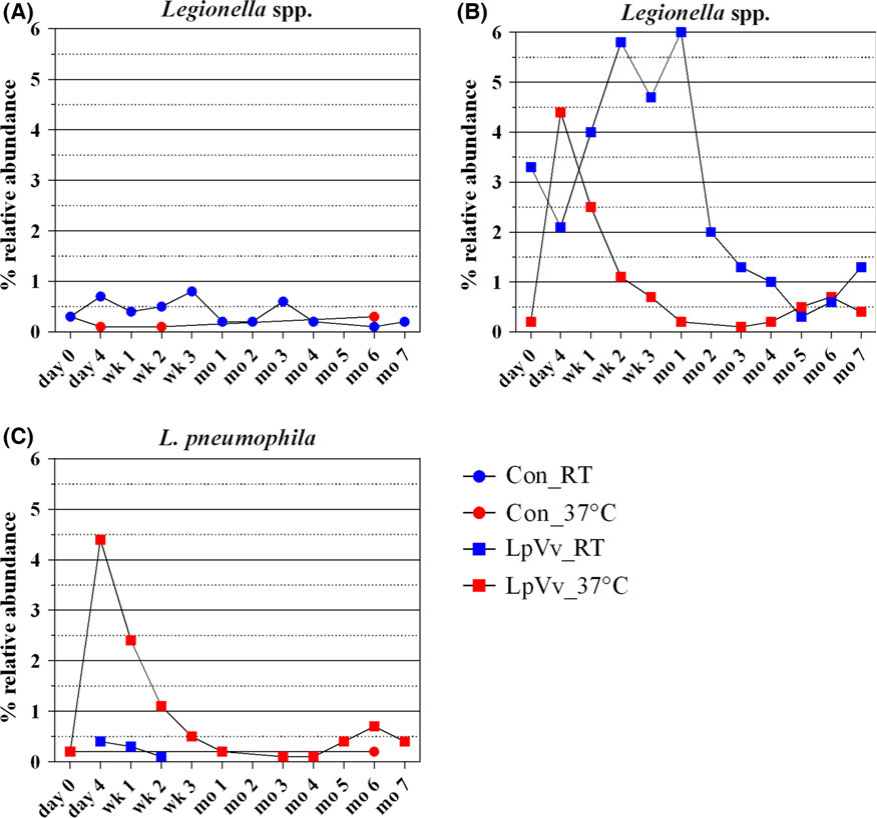
Relative abundance of *Legionella* spp.‐ and *L. pneumophila*‐associated sequences in biofilm samples. The relative abundance of *Legionella* spp. (A and B) and *L. pneumophila* (C) OTU taxonomic assignments is shown based on 16S rRNA gene sequences derived from Con_RT (blue circle), Con_37°C (red circle), LpVv_RT (blue square) and LpVv_37°C (red square) Cu DW biofilms at each time point.

All control (Con) and LpVv reactor biofilm samples contained levels of *Legionella* spp. detectable by quantitative PCR (qPCR; Fig. 1A and B respectively). *Legionella* spp. were consistently detected in all Cu biofilms from Con_RT reactors, ranging 1.9–2.9 log_10_ cell equivalents (CE) cm^−2^ from day 0 (before LpVv inoculation) to month 7 (except month 5, where levels dropped to 1.1 log_10_ CE cm^−2^; Fig. 1A, blue circles). In contrast, *Legionella* spp. were detected only at day 0 to week 1, month 2, month 6 and month 7, with levels between 1.2 and 1.7 log_10_ CE cm^−2^, with a spike at month 6 of 2.7 log_10_ CE cm^−2^ in Con_37°C biofilms (Fig. 1A, red circles). Notably, after the introduction of LpVv co‐cultures into the reactors (E2; Fig. S1), *Legionella* spp. were detected in all biofilm samples at each temperature, with LpVv_RT biofilm samples displaying a more consistent *Legionella* spp. level of between 2.8 and 4.3 log_10_ CE cm^−2^ for all time points, while levels within the LpVv_37°C‐derived biofilms fluctuated between 1.2 and 4.2 log_10_ CE cm^−2^ throughout the experiment (Fig. 1B). These observations based on qPCR were corroborated by the relative abundance of *Legionella* spp.‐associated sequences within the biofilm samples from each reactor (Fig. 2).

*Legionella pneumophila* was not detected in control reactor biofilm samples as measured by qPCR (except at month 6 where duplicate biofilm samples contained Lp levels between 2.4 and 3.0 log_10_ CE cm^−2^; Fig. 1C, red circles, black arrow). *Legionella pneumophila* levels in LpVv_RT biofilm samples were transient, detectable only between day 4 and month 1, with levels decreasing steadily post‐inoculation from 3.2 to 1.5 log_10_ CE cm^−2^ (Fig. 1C, blue squares). Furthermore, Lp levels in LpVv_RT biofilms were most likely due to the LpVv co‐culture inoculation as no Lp was detected at day 0 (before LpVv inoculation), which is in contrast to Lp levels in the LpVv_37°C biofilms, where starting Lp levels at day 0 (before LpVv inoculation) were 2.2 log_10_ CE cm^−2^ (Fig. 1C, red squares). Lp levels in LpVv_37°C biofilms proceeded to fluctuate between 1.2 and 4.2 log_10_ CE cm^−2^ in a similar pattern as observed for *Legionella* spp. levels in the same samples (Fig. 1B and C, red squares). Again, these qPCR‐based observations were corroborated by the relative abundance of *Legionella* spp.‐associated sequences within the biofilm samples from each reactor (Fig. 2). It has been previously demonstrated that partial 16S rRNA gene sequencing can identify *Legionella* isolates to the genus level and also allow for the differentiation of *L. pneumophila* from non‐pneumophila *Legionella* species (Wilson *et al*., 2007). Thus, in this study, the ability of the partial 16S rRNA gene sequences to differentiate *L. pneumophila* from non‐pneumophila *Legionella* species was confirmed by comparing sequences against the ribosomal databases SILVA, Greengenes and RDP, using 100% cut‐off and similarity values for the analysis which yielded consistent results for each database.

Notably, Vv was not detected in any of the control biofilm samples, and in inoculated reactors, Vv was detected only transiently in the LpVv_37°C biofilms at day 4 and week 1 (Fig. 1D, red squares, 2.7 and 2.3 log_10_ CE cm^−2^ respectively). In contrast, Vv levels were consistently and persistently high in LpVv_RT biofilms (Fig. 1D, blue squares; 2.4–3.1 log_10_ CE cm^−2^).

### Bacterial diversity and relative abundance in Cu DW biofilms

**Figure 3:**
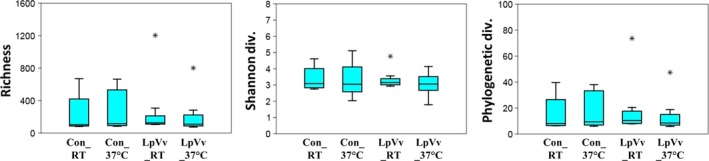
Alpha diversity indices for sequences derived from biofilm samples. Richness, Shannon diversity and phylogenetic diversity indices are shown for all biofilm‐derived sequences from each of the four reactors. The black dots represent libraries that were considered outliers during diversity analyses (LpVv_RT: month 5 and LpVv_37°C: week 1 samples).

Bacterial phylum‐level taxonomic assignments revealed different compositions within each of the four reactors with members of the phylum *Proteobacteria* representing the dominant population within biofilm samples (Table 1). Specifically, the relative abundance of α‐*Proteobacteria* in each reactor ranged from 41% to 57%; however, β‐*Proteobacteria* were about twice as abundant in biofilms incubated at RT (18–19%) than at 37°C (9–11%) while, conversely, γ‐*Proteobacteria* were about twice as abundant in biofilm incubated at 37°C (10–15%) than at RT (5–10%; Table 1). Notably, when compared to the other three reactors, *Acidobacteria*‐ and *Bacteroidetes*‐associated sequences were more dominant in LpVv_37°C (21 versus 0.1%) and LpVv_RT (11 versus 2–5%) respectively, indicating that, in the presence of Lp and Vv, higher temperatures supported a greater abundance of the *Acidobacteria* sequences while an abundance of *Bacteroidetes* bacteria‐associated sequences was more supported at lower temperatures. In Con_37°C biofilms, the absence of LpVv and higher temperatures seemed to support a higher diversity of bacteria including *Gemmatimonadetes*,* Planctomycetes* and δ‐*Proteobacteria* sequences being more abundant compared to samples from the other three reactors (Table 1). Moreover, introduction of LpVv into Cu DW biofilms seemed to result in a slight decrease in the group of bacterial operational taxonomic units (OTUs) that were detected at < 1% each (Table 1; LpVv samples 3–5% versus Con samples 6–7%), an observation that is corroborated by the decrease in richness and diversity indices of the LpVv biofilm samples (Fig. 3).

**Table 1:** Distribution of bacterial phyla within samples from each reactor

Phylum	Relative abundance (%)			
	Con_RT	Con_37°C	LpVv_RT	LpVv_37°C
*Proteobacteria*	79.0	74.9	81.3	62.1
α‐*Proteobacteria*	50.7	48.5	56.6	40.9
β‐*Proteobacteria*	18.0	9.1	19.4	10.7
γ‐*Proteobacteria*	9.6	15.1	4.9	10.0
δ‐*Proteobacteria*	0.7	2.2	0.4	0.4
*Actinobacteria*	9.0	8.6	3.5	6.8
*Acidobacteria*	0.1	0.1	0.1	21.2
*Bacteroidetes*	4.9	2.0	10.8	2.0
*Gemmatimonadetes*	0.5	4.1	0.6	1.9
*Planctomycetes*	0.4	3.1	0.4	1.4
Others < 1%	6.2	7.3	3.4	4.7
Total	100.0	100.0	100.0	100.0

Relative abundance is shown for each reactor for all time points.

Classes of *Proteobacteria* and their relative abundance are shown in grey text.

### Effect of temperature and LpVv inoculation on the microbiome of Cu DW biofilms

**Figure 4:**
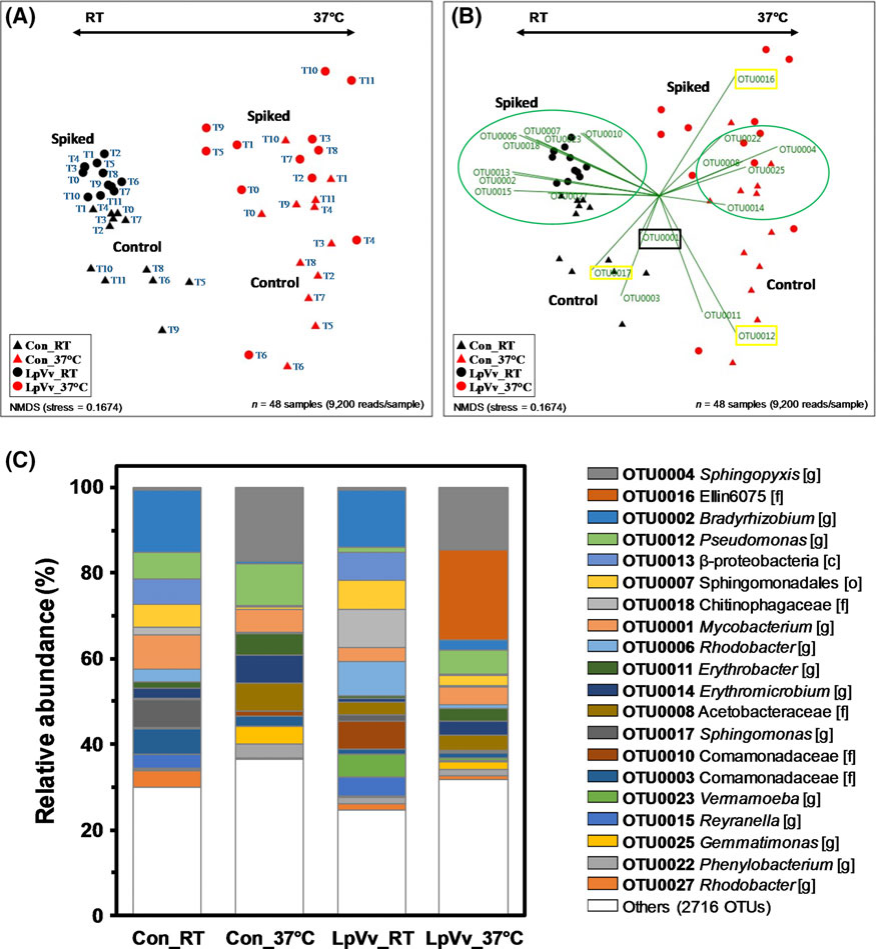
Non‐metric multidimensional scaling (NMDS) ordination plots and major OTU contributors for biofilm samples. A. NMDS plot representing the relationship for each reactor (symbols for each shown in the lower right‐hand corner square) between incubation temperature (red versus black symbols) and time points biofilms were sampled (T0–T11) with duplicates combined (*n* = 48). B. Green arrows indicate the orientation and contribution (i.e. greatest change) of OTUs between all the biofilm samples from each reactor. Yellow boxes indicate OTUs specific to a single reactor, and the black box indicates the OTU that is found in all reactor samples. OTUs that are found more abundantly in RT and 37 C samples are highlighted in the upper left and right ovals respectively. The distribution and taxonomy of the major OTU contributors are shown in (C).

Compared to Con biofilm samples at either temperature, there was an association between LpVv inoculation and a lower number of species (richness index: LpVv samples mean *c*. 180 versus Con samples mean *c*. 325) and reduced diversity of the bacterial community (Shannon and phylogenetic diversity indices: LpVv samples mean *c*. 3.3 and 11 versus Con samples mean *c*. 3.6 and 20 respectively; Fig. 3). Although LpVv introduction into the biofilm samples influenced overall community composition, principal coordinate analysis (PCoA) of the biofilm sequences indicated that temperature was the major driver of diversity, contributing to 31% of the total variation in biofilm samples at all time points compared to LpVv inoculation contributing 11% of the total variation (Fig. S2). Two‐way PERMANOVA of the samples also indicated that temperature played a major role in biofilm microbiome composition (*F* = 20.6, *P* = 0.0001) compared to pathogen and host inoculation (*F* = 6.1, *P* = 0.0001), while the combined effect of temperature and LpVv inoculation was also statistically relevant in determining overall diversity (*F* = 5.1, *P* = 0.0002; Table 2). A non‐metric multidimensional scaling (NMDS) ordination plot also indicated temperature had a more significant impact on the microbiomes (observed as the tight clustering of RT compared to 37°C biofilm samples) than LpVv inoculation (observed as individual reactor samples forming secondary clusters; Fig. 4A). Despite the relatively high OTU number identified from all biofilm samples in this study (> 2,700), only 20 OTUs were responsible for *c*. 66% of dissimilarity (based on SIMPER analysis) between biofilm samples (Fig. 4B and C).

**Table 2:** Results of two‐way PERMANOVA with permutation *N*: 9999

Source	Sum of squares	df	Mean square	*F*	*P*
Temperature					
RT versus 37°C	2.333	1.0	2.333	20.639	0.0001
Variable					
Con versus LpVv	0.690	1.0	0.690	6.103	0.0001
Interaction					
Temperature versus Variable	0.574	1.0	0.574	5.080	0.0002
Residual	4.974	44.0	0.113		
Total	8.572	47.0			

Nine of the 20 major OTUs identified were more abundant in Con_RT and LpVv_RT biofilm samples than in 37°C‐derived biofilms: class β‐*Proteobacteria*, order *Sphingomonadales*, family *Chitinophagaceae* and *Comamonadaceae* (two OTUs), and genus *Bradyrhizobium*,* Rhodobacter* (two OTUs) and *Reyranella* (Fig. 4B, upper left oval cluster, and C). Five of the 20 major OTUs identified were more abundant in Con_37°C and LpVv_37°C biofilm samples than in RT‐derived biofilms: family *Acetobacteraceae* and genus *Erythromicrobium*,* Gemmatimonas*,* Phenylobacterium* and *Sphingopyxis* (Fig. 4B, upper right oval cluster, and C). Unique OTUs, which were defined as being abundant in biofilm samples from a single reactor, included sequences associated with the genus *Sphingomonas* in Con_RT samples, *Pseudomonas* in Con_37°C and family Ellin6075 in LpVv_37°C samples (Fig. 4B, yellow boxes, and C). Ellin6075, affiliated with the bacterial phylum *Acidobacteria*, had a relative abundance of 21% in LpVv_37°C samples, but was not identified in the other three reactors (Fig. 4C), and could also be contributing exclusively to the relative abundance of 21% of *Acidobacteria* bacterial phylum sequences observed for LpVv_37°C samples (Table 1). Notably, a relatively high abundance (3–8%) of *Mycobacterium*‐associated sequences was found in all reactors and they did not cluster with any particular reactor samples in the NMDS plots indicating the organism's ability to stably colonize DW Cu biofilms regardless of temperature and pathogen/host colonization (Fig. 4B, black box, and C).

**Figure 5:**
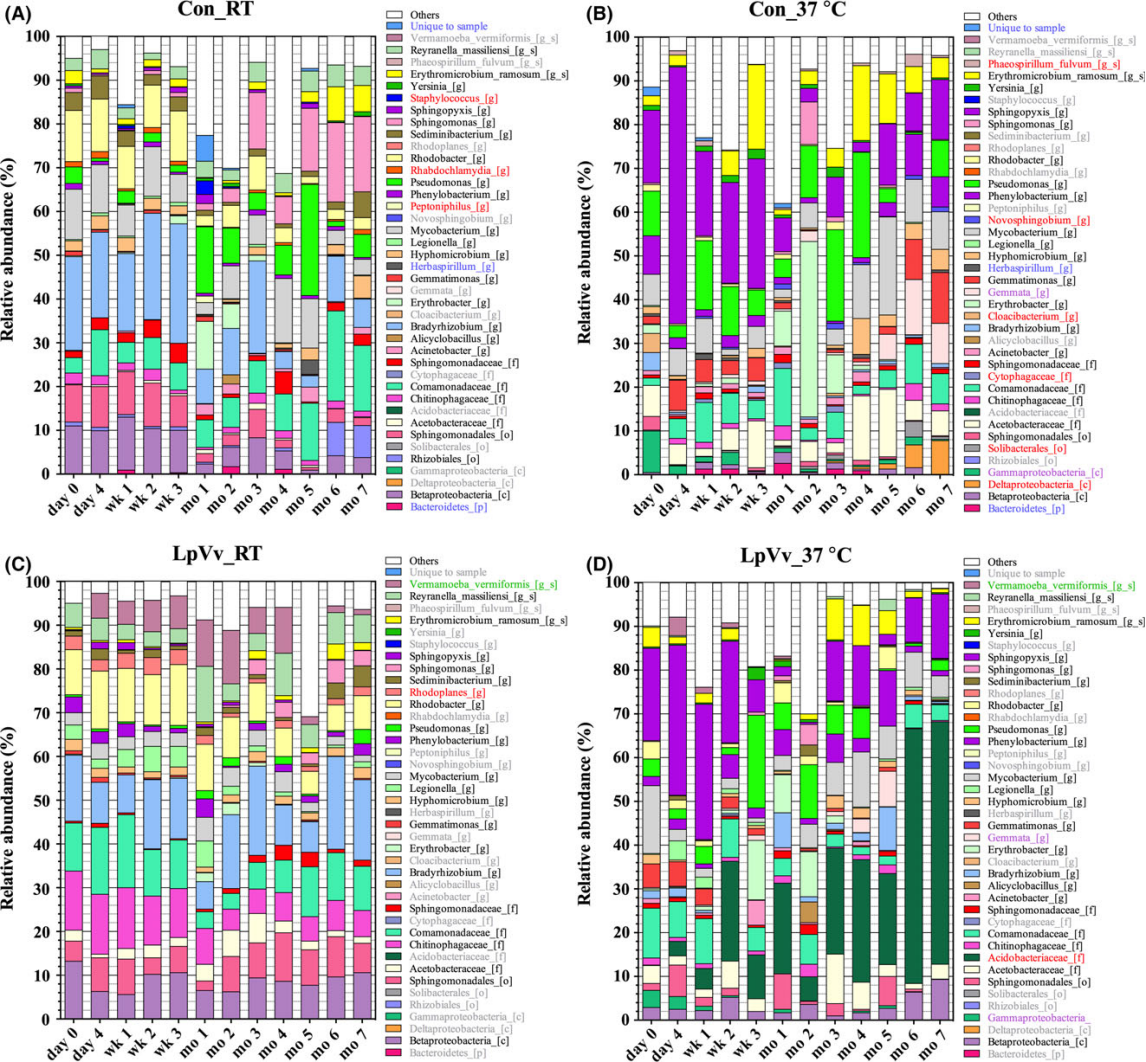
OTU taxonomic assignments expressed as percentage of total assigned sequences for each sample. 16S rRNA gene sequences derived from Con_RT (A), Con_37°C (B), LpVv_RT (C) and LpVv_37°C (D) Cu DW biofilms for each time point. In each legend, OTUs in coloured text indicate the following: absence or low abundance of sequences (grey); unique or abundant sequences for that reactor (red); sequences found in abundance in Con reactors only (blue); sequences found in abundance in LpVv reactors only (green); and sequences found in abundance in reactors incubated at 37°C only (purple).

To visualize community changes under the respective inoculation and temperature conditions over time, taxonomic assignments expressed as a percentage of total OTUs for each biofilm samples were plotted (Fig. 5). As stated above, temperature was the primary driver, followed by LpVv inoculation as the secondary driver, of microbiome diversity, which was visualized in the OTU profiles between the samples within each reactor (Fig. 5). The temporal OTU profiles revealed that not only were *Acidobacteriaceae* (Ellin6075)‐associated sequences highly abundant in LpVv_37°C samples, but abundance increased with time after inoculation (Fig. 5D, dark green bars). Moreover, starting at month 1, OTU profiles substantially shifted in each reactor as compared to their respective day 0–week 3 profiles, most notably for sequences associated with the majority of the top 20 contributing OTUs, in addition to sequences associated with the genera *Gemmata* in Con_37°C and LpVv_37°C samples and *Rhodoplanes* in LpVv_RT samples (Fig. 5). The change in OTU profiles after 1 month may also be explained by several significant changes in water quality characteristics over time such as an increase in per cent dissolved oxygen (DO) and decreases in alkalinity, barium (Ba) and sulfur (S), and fluctuations in aluminium (Al), calcium (Ca), sodium (Na), sulfur (S) and zinc (Zn) concentrations (Table 3). For example, percent DO at 6–7 months were significantly higher than the levels at day 4 to month 4 (ANOVA, *P* < 0.05), while Ca, Na, S and Zn levels fluctuated significantly throughout the 510‐day biofilm accumulation stage up until the 7‐month post‐inoculation time point (Table 3).

**Table 3:** Water quality measurements of CDC biofilm reactor feed water

Parameters	Units	Start to E1 (510 days)	E1 to E2 (101 days) T0	Post‐inoculation (time point[s])						
				1 month (T1–T5)	2 months (T6)	3 months (T7)	4 months (T8)	5 months (T9)	6 months (T10)	7 months (T11)
pH		8.43 ± 0.16	8.25 ± 0.37	8.45 ± 0.18	8.58 ± 0.05	8.57 ± 0.06	8.65 ± 0.06	8.55 ± 0.05	8.38 ± 0.05	8.38 ± 0.03
Temperature	°C	19.84 ± 0.71	20.60 ± 0.52	20.61 ± 0.38	20.37 ± 0.21	19.89 ± 0.60	18.40 ± 0.80	18.61 ± 0.78	18.89 ± 0.81	18.67 ± 0.34
Conductivity	mS cm^−1^	0.37 ± 0.08	0.365 ± 0.02	0.345 ± 0.02	0.395 ± 0.02	0.426 ± 0.01	0.455 ± 0.05	0.473 ± 0.05	0.322 ± 0.01	0.364 ± 0.01
DO	%	78.1 ± 8.2	69.7 ± 8.5	65.4 ± 2.4	68.4 ± 3.2	67.7 ± 2.3	72.5 ± 2.4^a^	75.7 ± 2.6^a^^,^ b ^,^ c	81.4 ± 1.8^a^^,^ b ^,^ c ^,^ d	82.8 ± 2.5^a^^,^ b ^,^ c ^,^ d
Free Cl_2_	mg l^−1^	0.40 ± 0.23	0.44 ± 0.07	0.49 ± 0.08	0.61 ± 0.05	0.60 ± 0.05	0.68 ± 0.14	0.59 ± 0.12	0.34 ± 0.12	0.41 ± 0.06
Total Cl_2_		0.44 ± 0.22	0.47 ± 0.07	0.609 ± 0.05	0.637 ± 0.04	0.628 ± 0.04	0.714 ± 0.14	0.633 ± 0.11	0.391 ± 0.12	0.458 ± 0.06
Alkalinity		68.6 ± 9.8	68.5 ± 3.9	78.7 ± 6.2	79.6 ± 5.2	76.8 ± 0.8	83.0 ± 6.0^c^	87.8 ± 5.1	58.6 ± 2.3^a^^,^ b ^,^ c ^,^ d ^,^ e	63.3 ± 2.4^a^^,^ b ^,^ c ^,^ d ^,^ e
TOC		1.02 ± 0.41	0.96 ± 0.42	1.02 ± 0.30	0.68 ± 0.26	0.54 ± 0.03	0.86 ± 0.15	1.01 ± 0.15	1.07 ± 0.10	0.92 ± 0.07
NO_3_		0.82 ± 0.17	0.84 ± 0.18	0.88 ± 0.14	0.72 ± 0.04	0.73 ± 0.03	0.89 ± 0.11	0.92 ± 0.09	0.87 ± 0.13	0.97 ± 0.05
PO_4_		0.22 ± 0.06	0.19 ± 0.04	0.21 ± 0.04	0.22 ± 0.02	0.15 ± 0.05	0.15 ± 0.04	0.15 ± 0.02	0.10 ± 0.02	0.11 ± 0.03
Ca		31.1 ± 3.0	28.9 ± 1.0	28.89 ± 1.8	32.01 ± 1.9	34.09 ± 1.8	35.63 ± 5.1^a^	37.96 ± 2.8	27.34 ± 1.1^c^^,^ d ^,^ e	30.4 ± 1.3
K		2.3 ± 0.5	2.0 ± 0.9	2.5 ± 0.2	2.6 ± 0.1	2.6 ± 0.1	3.0 ± 0.4	3.2 ± 0.3	2.2 ± 0.1	1.9 ± 0.1
Mg		10.0 ± 2.0	9.3 ± 0.6	8.4 ± 0.7	9.9 ± 0.5	10.3 ± 0.5	11.4 ± 1.3	12.2 ± 1.0	8.1 ± 0.4	9.1 ± 0.5
Na		24.6 ± 8.4	23.1 ± 2.1	19.1 ± 1.1	24.7 ± 1.9	28.8 ± 1.4^a^	30.9 ± 4.9^a^^,^ b	32.2 ± 5.1^a^	19.2 ± 1.4^c^^,^ d ^,^ e	22.5 ± 1.5^d^
P		0.16 ± 0.03	0.17 ± 0.08	0.17 ± 0.01	0.17 ± 0.01	0.16 ± 0.01	0.16 ± 0.01	0.17 ± 0.01	0.13 ± 0.01	0.13 ± 0.01
S		23.3 ± 5.7	21.2 ± 2.1	17.7 ± 0.6	22.2 ± 1.6	26.7 ± 2.2^a^	27.0 ± 4.2^a^	27.9 ± 3.0^a^	17.9 ± 1.3^c^^,^ d ^,^ e	19.5 ± 0.8^c^^,^ d
Si		2.2 ± 0.7	2.1 ± 0.3	3.0 ± 0.1	2.6 ± 0.1	2.3 ± 0.1	2.3 ± 0.0	2.2 ± 0.1	2.8 ± 0.2	2.9 ± 0.1
Sr		0.22 ± 0.06	0.22 ± 0.02	0.19 ± 0.01	0.22 ± 0.01	0.23 ± 0.02	0.28 ± 0.02	0.28 ± 0.04	0.17 ± 0.01	0.19 ± 0.01
Al	μg l^−1^	45.9 ± 20.0	54.1 ± 13.9	67.2 ± 7.1	74.2 ± 11.6^a^	61.6 ± 11.2^b^	42.4 ± 4.9^a^^,^ b ^,^ c	45.4 ± 3.3^a^^,^ b ^,^ c	41.5 ± 2.2^b^^,^ c	31.0 ± 5.1^a^^,^ b ^,^ c ^,^ d ^,^ e ^,^ f
Ba		38.6 ± 7.4	36.7 ± 4.2	35.9 ± 1.5	41.1 ± 2.1	43.6 ± 1.5^a^	42.3 ± 3.8^a^	42.4 ± 4.6	33.2 ± 1.5^b^^,^ c ^,^ d	32.3 ± 1.3^b^^,^ c ^,^ d
Cu		26.0 ± 4.9	20.7 ± 2.2	17.8 ± 0.8	17.7 ± 1.2	16.0 ± 0.9	16.6 ± 1.8	19.9 ± 0.9	19.0 ± 1.1	21.2 ± 2.3
Fe		21.2 ± 24	14.7 ± 4.0	11.9 ± 2.8	16.6 ± 5.9	14.9 ± 1.4	16.9 ± 6.9	14.6 ± 1.5	15.2 ± 1.5	12.6 ± 10.9
Pb		3.3 ± 2.2	2.4 ± 1.1	3.6 ± 0.8	2.3 ± 1.6	1.3 ± 1.5	2.3 ± 0.3	2.2 ± 0.2	1.8 ± 1	3.3 ± 2.2
Zn		89.8 ± 25.3	90.3 ± 12.0	66.1 ± 4.3	73.7 ± 4.9^a^	80.7 ± 5.4^a^^,^ b	84.6 ± 12.9^a^^,^ b	113.8 ± 21.6^a^^,^ b ^,^ c ^,^ d	132.5 ± 6.5^b^^,^ c ^,^ d ^,^ e	141.6 ± 7.6^a^^,^ b ^,^ c ^,^ d ^,^ e ^,^ f

Total organic carbon, TOC; nitrate, NO_3_; calcium, Ca; potassium, K: magnesium, Mg; sodium, Na; sulfur, S; silicon, Si; phosphate, PO_4_; aluminium, Al; borium, Ba; copper, Cu; iron, Fe; phosphorous, P; lead, Pb; strontium, Sr; zinc, Zn; alkalinity as measured by CaCO_3_ concentration.

Values expressed as mean ± standard deviation.

Two‐way ANOVA for each parameter: ^a^ *P* < 0.05 for T1‐T5 versus T6, T7, T8, T9, T10 and T11. ^b^ *P* < 0.05 for T6 versus T7, T8, T9, T10 and T11. ^c^ *P* < 0.05 for T7 versus T8, T9, T10 and T11. ^d^ *P* < 0.01 for T8 versus T9, T10 and T11. ^e^ *P* < 0.05 for T9 versus T10 and T11. ^f^ *P* < 0.05 for T10 versus T11.

## Discussion

### Impact of *L. pneumophila* colonization within copper DW biofilms

Copper (Cu) is a commonly used pipe material within premise plumbing systems (Council, 2006). The antimicrobial properties of Cu on *L. pneumophila* inactivation have been demonstrated previously (Lin *et al*., 1996; Biurrun *et al*., 1999), with the combined effects of copper–silver ionization considered an effective control strategy for *L. pneumophila* in US hospital water systems (Lin *et al*., 2011). However, it has been reported that the antimicrobial properties of Cu can be negatively impacted by biofilm age, accumulation and presence of corrosion products. As measured by total bacterial counts, early biofilm growth (≤ 30 days) on Cu surfaces was lower than on PVC or PEX material (Mathys *et al*., 2008; Morvay *et al*., 2011), while in older, mature biofilms (18 months old), Cu biofilm growth was two‐ to eightfold higher than PVC, PE and SS biofilms (Wingender and Flemming, 2004). Moreover, *Legionella* densities were initially higher in < 250‐day‐old SS than in Cu biofilms, but after 24 months, similar levels of *Legionella* were observed within SS and Cu biofilms suggesting the inhibitory effect of Cu on *Legionella* may be transient and reduced by the accumulation of corrosion products on the Cu surfaces over time (van der Kooij *et al*., 2005). Although there is currently no standard definition of a mature DW biofilm (with studies labelling 1‐ to 2‐year‐old (Boe‐Hansen *et al*., 2002; Wingender and Flemming, 2004) and 20‐year‐old (Henne *et al*., 2012) DW biofilms as mature), the consensus seems to be that mature biofilms consist of a complex and functional microbial community that exhibits physiological cooperation and metabolic efficiency all of which is gradually developed (Costerton *et al*., 1995; Dunne, 2002). Thus, mature DW biofilms may be a more accurate representation of biofilms within building systems, and hence more relevant in examining *Legionella* colonization in premise plumbing systems.

Using approximately 20‐month‐old biofilms, the results from this study indicated that Cu DW biofilms inoculated with LpVv co‐cultures exhibited temperature‐dependent colonization of Lp at high and persistent levels for up to 7 months (Fig. 1C). Specifically, colonization of Lp within Cu biofilms incubated at RT resulted in an increase in the overall levels of *Legionella* spp. members; yet colonization of Lp within biofilms incubated at 37°C resulted in the dominance of Lp over other *Legionella* spp. Non‐*L. pneumophila* members of the *Legionella* genus seem to be more adept at lower temperature growth (Figs 1A and 2), while higher temperatures appear to select for Lp growth, an observation which has been previously reported (Wullings and van der Kooij, 2006). Thus, although the temperature‐dependent growth of Lp in DW has been described in other studies (Rogers *et al*., 1994; Rhoads *et al*., 2015), an interesting observation from this study was the influence of Lp biofilm colonization on other *Legionella* genus members, which had not been directly demonstrated previously. Additionally, this study further corroborates the general observation that Cu pipe is not universally antimicrobial against *Legionella* spp. nor *L. pneumophila*, especially at the higher pH values (> pH 8.2; Table 3; Lin 2002) tested in the current study.

### Effect of *V. vermiformis* inoculation into biofilms

Forty‐eight‐hour co‐culture of Lp and its FLA host, Vv, was introduced into biofilms based on previous studies indicating upregulation of Lp virulence after intra‐amoeba passage and during their co‐inoculations into mice (Brieland *et al*., 1996, 1997). Thus, it was hypothesized that increased colonization of DW biofilms could be a function of phenotypic changes induced during Lp passage and co‐culture with Vv hosts, which was exploited in this study. While Vv levels were relatively high and persistent within LpVv_RT biofilms, Lp levels in the same samples steadily decreased from day 4 to month 1, indicating parasitization and interactions with Vv did not sustain Lp levels at RT (Fig. 1C and D). This is supported by previous data stating that at < 20°C, *L. pneumophila* was actively digested and/or eliminated from the FLA, *Acanthamoeba polyphaga*, while at temperature above 25°C, the amoebae was parasitized by Lp (Ohno *et al*., 2008). Moreover, Vv cells were not detected past 1 week post‐inoculation at 37°C (Fig. 1D), while persistent Lp levels were observed in the LpVv_37°C biofilms (Fig. 1C), suggesting Lp biofilm colonization at this temperature was most likely not due to the presence of Vv cells. The qPCR detection of Lp only at month 6 (Fig. 1C, black arrow) and identification of Lp‐associated sequences at day 0, before LpVv inoculation and month 6 (Fig. 2B, red circle; 0.2% relative abundance) in Con_37°C biofilm samples may also be further indication that this temperature was amenable to Lp biofilm growth independent of Vv interactions. These results suggest that stable colonization of Vv within Cu DW biofilms may be strongly driven by temperature; however, the complete, initial parasitization of Vv cells at 37°C by Lp bacteria (and potentially other endemic biofilm amoebae) and their effect on Lp throughout the rest of the experiment cannot be excluded. Additionally, contrary to previous reports indicating the abundance of Vv in both chlorinated and chloraminated DW systems (Thomas *et al*., 2006; Corsaro *et al*., 2010; Valster *et al*., 2011; Wang *et al*., 2012), as well as within the building system used in this study (Buse *et al*., 2013), Vv cells were not detected in any of the control biofilm samples, which could indicate a preference for the bulk water phase and a need for high cellular densities for stable and persistent biofilm colonization.

### Temperature and colonization of *L. pneumophila* and *V. vermiformis* are major drivers for overall biofilm community composition

Principal coordinate analysis, two‐way PERMANOVA, and NMDS analyses all indicated that temperature was the major driver of biofilm microbiome composition followed by LpVv colonization (Fig. S2, Table 2, and Fig. 3A, respectively). The communities inoculated with LpVv at RT and 37°C maintained a more stable composition, compared to control biofilms, based on their lower coefficient of variation as indicated in Figs 3 and 4B. FLA grazing on stream water‐derived biofilms and *Klebsiella pneumoniae*,* Pseudomonas fluorescens* and *Staphylococcus epidermidis* biofilms were shown to alter overall morphology and microbial composition of their respective biofilms (Huws *et al*., 2005; Böhme *et al*., 2009). However, in this study, the predatory impact of Vv biofilm colonization on the bacterial community was not clear from the bacterial OTU distribution data especially given the fact that the actively feeding, trophozoite or metabolically dormant cyst forms cannot be distinguished using qPCR or sequencing analyses, nor were other amoebae investigated in this study. It must be noted that OTU 0023 was identified as Vv‐associated sequences (Figs 4C and 5C and D, green highlighted text), yet only 16S rRNA gene amplification was performed. Thus, the Vv‐associated sequences could have been derived either from intracellular bacterial organisms previously associated with Vv cells or from mitochondrial Vv sequences that were not taxonomically labelled as such and thus were not removed by mothur. This OTU was left in the analysis as it was one of the top 20 major OTU indicators of microbial composition (high relative abundance in LpVv_RT samples; Fig. 4C) and was previously associated with bacterial sequences derived from two different sources of groundwater (Pedersen *et al*., 1997; Benzine *et al*., 2013).

Notably, *Mycobacterium avium*,* M. intracellulare* and *M. abscessus*, other opportunistic water‐based pathogens, have been shown to readily adhere and stably colonize DW biofilms grown on glass, stainless steel, PVC and zinc‐galvanized steel coupons (Mullis and Falkinham, 2013). Their stable colonization and relatively high abundance within DW biofilms were supported in this study, for Cu surfaces, as *Mycobacterium* was found to be one of the top 20 indicator OTUs impacting overall diversity with relative abundance across all samples unaffected by temperature or LpVv colonization (Fig. 4C).

Although the water quality parameters collected weekly in this study corresponded to the influent water, and not water collected from within each reactor, several parameters that were shown to change significantly over the study period have been previously correlated with *Legionella* occurrence and abundance within DW systems [reviewed in Buse *et al*., 2012]. Specifically, DW containing > 50 μg Cu l^−1^, < 100 μg l^−1^ Zn, < 20 μg l^−1^ Fe and < 6 μg l^−1^ Mn was found to be protective against legionellae colonization, while in hot DW samples, the measured mean levels of DO (66%), TOC (6 ppm), Ca (25 mg l^−1^), sulfate (33 mg l^−1^) and nitrate (1 mg l^−1^) were positively correlated with *Legionella* densities (Buse *et al*., 2012). Moreover, variations in bulk DW community profiles have been associated with water quality parameters such as concentrations of disinfectant, phosphorus, sulfate and Mg (Ji *et al*., 2015). Thus, significant changes in the various influent water quality parameters measured in this study (Table 3), in concert with LpVv (and other possible micro‐eukaryotes) colonization, could have influenced biofilm microbiome composition throughout the study period. However, no statistical correlations could be drawn, as influent water was analysed rather than water collected from each CDC reactor; thus, future research will aim to address the putative importance of water quality on the biofilm microbiome.

Illumina sequencing of both bulk water and biofilm bacterial communities derived from chlorinated and chloraminated treated water was previously shown to be dominated by the phyla *Acidobacteria*,* Actinobacteria*,* Bacteroidetes*,* Gemmatimonadetes*,* Planctomyces* and *Proteobacteria* (Pinto *et al*., 2012; Buse *et al*., 2014; Gomez‐Alvarez *et al*., 2014), which was similar to the results obtained in this study (Table 1). Compared to earlier DNA sequencing methods, such as Sanger‐based sequencing, there are an increasing number of studies utilizing next‐generation sequencing technology to improve sequencing depth to better characterize the complex microbial composition of DW biofilms. Nonetheless, both methods have been able to identify sequences associated with the genera *Legionella*,* Mycobacteria* and/or *Pseudomonas* within chlorinated and chloraminated DW‐derived biofilms grown on concrete/steel, Cu, cast iron, polycarbonate, PVC and SS surfaces (McBain *et al*., 2003; Zhang *et al*., 2012; Lin *et al*., 2013; Gomez‐Alvarez *et al*., 2014; Chao *et al*., 2015) indicating that DW biofilms may be a potential and continual source of environmental (water‐based) pathogens (Ashbolt, 2015).

### Conclusion

Collectively, this study demonstrated that *L. pneumophila* can colonize mature DW biofilms grown on copper surfaces at relatively high pH levels (> pH 8.2) in which the antimicrobial properties of copper can be negatively impacted. Once incorporated, *L. pneumophila* may persist up to 1 month at cold‐water temperatures for premise plumbing (20°C) and up to 7 months at higher temperatures (37°C), which may pose a significant public health risk due to the extended colonization potential of these pathogens within premise plumbing. Temperature and inoculation of *L. pneumophila* and *V. vermiformis* were strong drivers of biofilm microbiome diversity, with major changes in bacterial composition between 1 and 7 months, compared to those between day 0 and 1 month. Notably, the aged copper used in this study did not universally exclude *Legionella* spp. nor *L. pneumophila* colonization, and *V. vermiformis* was not a prerequisite for proliferation or persistence of *L. pneumophila*. Moreover, *L. pneumophila* seemed to influence the presence and abundance of other *Legionella* spp., as well as other genera within the biofilm microbiome, further underscoring the need to elucidate *L. pneumophila* behaviour and their ecological role within mature DW biofilms in premise plumbing.

## Experimental procedures

### Bacterial and amoebal co‐culture preparation

*Legionella pneumophila* strain Chicago‐2 (Lp) is a clinical isolate derived from a human lung (American Type Culture Collection [ATCC] 33215), while *V*. *vermiformis* strain CDC‐19 (Vv) is an environmental isolate derived from a hospital cooling tower drain (ATCC 50237). Lp and Vv were grown and enumerated as previously described (Buse and Ashbolt, 2011). Briefly, Lp was grown overnight with continuous shaking at 37°C in buffered yeast extract (BYE) broth and Vv cells were grown as monolayers at 30°C in ATCC 1034 medium. Lp and Vv cultures were washed and diluted to desired concentrations in Page's amoeba saline (PAS). Lp densities, as measured by colony‐forming units (CFU), were determined by taking a small aliquot of the bacterial suspension, performing serial dilutions, and plating on buffered charcoal yeast extract (BCYE) agar plates (BD Diagnostics, Franklin Lakes, NJ, USA) which were incubated for 48 h at 37°C. Vv densities were enumerated using light microscopy and an improved Neubauer hemocytometer (Hausser Scientific, Horsham, PA, USA).

Previous studies indicated that during Lp infection of Vv cells, Lp densities peaked after 48 h of co‐culture (Buse and Ashbolt, 2011). Thus, for Lp and Vv inoculation into Centers for Disease Control and Prevention (CDC) biofilm reactors (Biosurface Technologies Corp., Bozeman, MT, USA), co‐cultures were prepared by washing overnight cultures of Lp and 7‐day‐old Vv cultures in PAS, co‐inoculating each into 75‐cm^2^ tissue culture flasks to generate a 15:1 bacteria‐to‐amoeba ratio (6.93 log_10_ CFU ml^−1^ of Lp and 5.74 log_10_ cells ml^−1^ of Vv), and incubating the flasks for 48 h at 32°C. After incubation, flasks were harvested and Lp and Vv enumerated as described above (7.27 log_10_ CFU ml^−1^ of Lp and 5.67 log_10_ cells ml^−1^ of Vv).

### CDC biofilm reactor set‐up and sample collection

Four CDC biofilm reactors containing copper (Cu) coupons (surface area of 1.27 cm^2^) were fed with ambient (19.8 ± 0.8°C) DW from a light‐protected storage tank (23 l) at 150 ml h^−1^ (2.7‐h hydraulic residence time) using a peristaltic pump and Norprene™ food‐grade tubing (Cole Parmer, Vernon Hills, IL, USA). The Cu coupons used in this study were made from C10100 alloy (99.99% Cu), which is similar in composition to the Cu alloy C12200 used in Cu plumbing (99.9% Cu and silver combined and 0.015–0.04% phosphorus as per requirements of the American Society for Testing and Materials B88 Standard Specification for Seamless Copper Water Tube). In‐premise flow and stagnation periods were simulated by placing reactors on a magnetic stir plate set at approximately 100–125 rpm, which was activated for 30 min every 2 h [start to event 1 (E1)] and then every 2 h during a 10‐h window on weekdays [E1 to the inoculation event 2 (E2); Fig. S1]. Mature DW biofilms were allowed to establish on the Cu coupon surfaces for 510 days under ambient/room temperature (RT) conditions. At E1, temperatures within two of the four reactors, which served as control/mock‐inoculated (Con) and Lp and Vv (LpVv)‐inoculated reactors, were elevated to 37°C for the duration of the experiment (labelled Con_37°C and LpVv_37°C, respectively) with the two remaining reactors, designated Con_RT and LpVv_RT, maintained at RT. DW biofilms were then allowed to adjust to the new temperature conditions for 101 days before LpVv inoculation (E2). Prior to E2, duplicate coupons from each reactor were collected to record established biofilm characteristics [time 0 (T0)] as a baseline comparison to post‐inoculation samples (T1–T11).

For LpVv co‐culture inoculations (E2), reactors were disconnected from the peristaltic feed pump, with effluent lines clamped shut, and placed in a biological safety cabinet where the total DW volume was removed (approximately 400 ml) and replaced with 368 ml of 0.22 μm filtered, autoclaved tap water (fatH_2_O). The control reactors, Con_RT and Con_37°C, were inoculated with 32 ml fatH_2_O, while the LpVv_RT and LpVv_37°C reactors were inoculated with 32 ml of the LpVv co‐culture suspension prepared as described above. Reactors were replaced onto the magnetic stir plates and incubated at either RT or 37°C. Microbial adhesion was allowed to occur for 24 h with 30 min of mixing every 2 h. After the 24‐h incubation, each reactor was connected back to the peristaltic feed pump with effluent clamps released to allow flow. Two coupons (replicate 1, R1; and replicate 2, R2) were removed from each reactor at day 4 (T1), week 1 (T2), week 2 (T3), week 3 (T4) and month 1 (T5), 2 (T6), 3 (T7), 4 (T8), 5 (T9), 6 (T10) and 7 (T11) post‐inoculation (Fig. S1). Biofilms were collected from coupon surfaces as previously described (Buse *et al*., 2013b; Buse *et al*., 2014). Briefly, a sterile wooden stick was used to scrape the surface and was then rinsed in 300 μl of fatH_2_O. The coupon surface was washed twice with 100 μl fatH_2_O resulting in a final volume of 500 μl suspended biofilm material.

### Water quality measurements

An aliquot of 250 ml of the tank water feeding the CDC reactors was sampled every week prior to and 7 months after Lp and Vv inoculation (*n* = 109 and 28 respectively). Water samples were analysed for free and total chlorine (Cl_2_), pH, temperature, conductivity, and per cent dissolved oxygen (DO; Table 3). The pH was measured using a glass electrode UltraBASIC‐10 meter (Denver Instrument, USA) and temperature, conductivity, and per cent dissolved oxygen using a YSI 566 Multi Probe System (YSI Environmental, Yellow Springs, OH, USA). Free and total Cl_2_ were measured using a TNT866 Chlorine Kit (Hach Company, Loveland, CO, USA) and the Hach^®^ DR2800 spectrophotometer (Hach Company). An additional 550 ml of tank water was also submitted for weekly water quality analysis to assay total organic carbon (TOC; EPA Method 415.3 rev1.1), trace metals Cu, Fe, Mg, P, and Zn (EPA Method 200.7), phosphate (PO4; EPA Method 365.1), and nitrate (NO3; EPA Method 353.2) by the National Risk Management Research Laboratory at the US Environmental Protection Agency in Cincinnati, OH (Table 3).

### DNA isolation and quantitative PCR

DNA was extracted from biofilm suspensions using the T&C buffer and MasterPure Complete DNA Purification Kit™ (Epicentre Biotechnologies, Madison, WI, USA) as described previously (Buse *et al*., 2014). Biofilm DNA samples were analysed using the Applied Biosystems 7900 HT Fast Real‐Time PCR System (Applied Biosystems, Foster City, CA, USA). Each sample was analysed in duplicate in a reaction mixture (20 μl final volume) containing the following: 1 × Power SYBR^®^ Green PCR Master Mix (Applied Biosystems); 500 nM of each forward and reverse Lp‐specific primers targeting the *sidF* gene (Lu *et al*., 2013) or Vv‐specific primers targeting the 18S rRNA gene Hv1227/1728R (Kuiper *et al*., 2006); and 5 g bovine serum albumin. For Lp, the qPCR conditions consisted of an initial denaturation step of 10 min at 95°C, followed by 40 cycles of 15 s at 95°C, 30 s at 64°C, and 30 s at 72°C with a final hold at 72°C for 2 min. For Vv, cycling conditions included pre‐denaturation at 95°C for 3 min, 40 cycles of 20 s at 95°C, 30 s at 56°C, and 40 s at 72°C with a final extension step at 72°C for 10 min. Fluorescent detection was performed at the annealing phase and during subsequent dissociation curve analysis to confirm that a single product had been amplified. TaqMan qPCR assay for *Legionella* spp. detection was performed using the 16S rRNA gene primers 16S‐LegF1c/16S‐LegR1c and probe 16S‐LegP1 (Lu *et al*., 2015). The cycling conditions consisted of a pre‐incubation step at 50°C for 2 min and a pre‐denaturation step at 95°C for 10 min, followed by 40 cycles of denaturation at 95°C for 10 s, annealing at 50°C for 30 s, and extension at 70°C for 30 s.

All qPCR assays were performed in a 96‐well plate containing DNA standards and no‐template controls. The threshold cycles (Ct) were calculated using the sequence detection systems software v 2.3 (Life Technologies, Carlsbad, CA, USA). The reaction efficiency was calculated using the following equation: efficiency % = 100 × (10(–1/slope) – 1) (Applied Biosystems). Experiments were performed with undiluted and 10‐fold‐diluted template DNA in duplicate to verify the absence of qPCR inhibition. Data were expressed as cell equivalents (CE), based on culture densities (as measured by colony‐forming units), which were used to generate standard curves against the surface area of each reactor coupon (CE cm^−2^). All standards were generated in triplicate and the best‐fit standard curve used in data analysis. The limit of quantification (LOQ) for each qPCR assay was 1 log_10_ CE cm^−2^.

### Illumina sequencing

Biofilm samples were subjected to PCR amplification using the universal bacterial/archaeal primer set F515 (5′ GTG CCA GCM GCC GCG GTA A 3′)/R806 (5′ GGA CTA CHV GGG TWT CTA AT 3′, barcoded, #0–95) targeting the V4 region of 16S rRNA gene, which was determined to yield optimal community clustering with this particular read length (Caporaso *et al*., 2011). Samples were prepared following the Earth Microbiome Project 16S rRNA Amplification Protocol version 4_13 (ftp://ftp.metagenomics.anl.gov/data/misc/EMP/SupplementaryFile1_barcoded_primers_515F_806R.txt) with the exceptions of using molecular biology grade water (Quality Biological, Gaithersburg, MD, USA) and the QIAquick PCR Purification Kit (Qiagen, Valencia, CA, USA). Of the 96 biofilm samples, 90 were pooled on an equal molar basis (200 ng), and the remaining six samples with low‐yield PCR products were pooled based on ‘maximum volume’ criteria (maximum volume of the 90 samples, which is 50 μl). Paired‐end 250‐cycle Illumina sequencing was performed by the Genomics Research Laboratory at the Virginia Bioinformatics Institute (Blacksburg, VA, USA).

### Sequence data analyses

Reads processing was performed using the software mothur v1.36.1 (https://www.mothur.org; Schloss *et al*., 2009) following the protocol of Kozich *et al*. (2013). Briefly, fastq files with forward and reverse reads were used to form contigs. Reads were screened and removed if they (i) had a length < 252 bp, (ii) contained ambiguous bases (Ns), (iii) contained homopolymers greater than seven bases, (iv) were identified as chimera, or (v) were classified as unknown, chloroplasts or mitochondria. Reads were aligned and grouped with 97% sequence identity as the cut‐off point for each operational taxonomic unit (OTU). Taxonomic classification was obtained using the Greengenes reference taxonomy database release gg_13_8_99. A total of 1 721 964 16S rRNA gene reads were analysed in this study. Prior to community analysis, samples were rarefied to the smallest data set of 9200 reads (Gihring *et al*., 2012). The Illumina reads have been deposited in the National Center for Biotechnology Information (NCBI) database BioProject number PRJNA214912: SRA accession #SAMN04528999‐SAMN04529093.

### Statistical analysis

Means and standard error means were calculated using duplicate samples collected for all biofilm and effluent water sample types at each time point. Statistical significance for water quality parameters was determined using ANOVA followed by the Tukey post‐test (prism 6; GraphPad Software, La Jolla, CA, USA). Alpha diversity indices (supplemental information) and PERMANOVA of Illumina sequences were calculated using past version 2.03 (http://folk.uio.no/ohammer/past/; Hammer *et al*., 2001). A non‐metric multidimensional scaling (NMDS) ordination plot based on the Jensen–Shannon distance (JSD) was also generated using the past software with robustness of the ordination plot evaluated for goodness of fit using the Shepard diagram (Wickelmaier, 2003).

## Conflict of Interest

None declared.

## Supporting information


**Fig. S1.** Biofilm reactor set‐up and experimental timeline. Four CDC biofilm reactors were operated as described in Materials and Methods (A). Drinking water biofilms were allowed to develop on the copper (Cu) surfaces within all four reactors (START) for 510 days under ambient/room temperature (RT) conditions (B) before any manipulation of the reactors occurred. At event 1 (E1), temperatures within two of the reactors, that would serve as the control, mock‐inoculated (Con) and *L. pneumophila* and *V. vermiformis* (LpVv)‐inoculated reactors, were elevated to 37°C for the duration of the experiment (Con_37°C and LpVv_37°C, respectively). Biofilms were allowed 101 days to adjust to the temperature change before any further manipulation of the reactors occurred. Prior to inoculation, biofilm material was collected from duplicate Cu coupons from each reactor at T0. After T0 sampling, event 2 (E2) consisted of inoculating 48 h co‐cultures of Lp and Vv into reactors incubated at RT and 37°C, LpVv_RT and LpVv_37°C, respectively. Control reactors, incubated at RT and 37°C, were inoculated with sterile, co‐culture buffer (Con_RT and Con_37°C respectively). Biofilm material was collected from duplicate Cu coupons from each reactor at the time points indicated (B).

**Fig. S2.** Principal Coordinate Analysis (PCoA) for sequences derived from biofilm samples. The PCoA graph indicates the contribution of temperature (*x*‐axis) and effect of inoculation (Con versus LpVv, *y*‐axis) on the overall variation of the biofilm reactor sequence libraries at each of the twelve time points.

**Appendix S1.** Diversity index formula and descriptions.
