# Supplementary figures and images for: Effect of temperature and colonization of Legionella pneumophila and Vermamoeba vermiformis on bacterial community composition of copper drinking water biofilms
Source: Microb Biotechnol. 2017 Jan 18;10(4):773–88. doi: 10.1111/1751-7915.12457 (PMC5481522; doi:10.1111/1751-7915.12457)

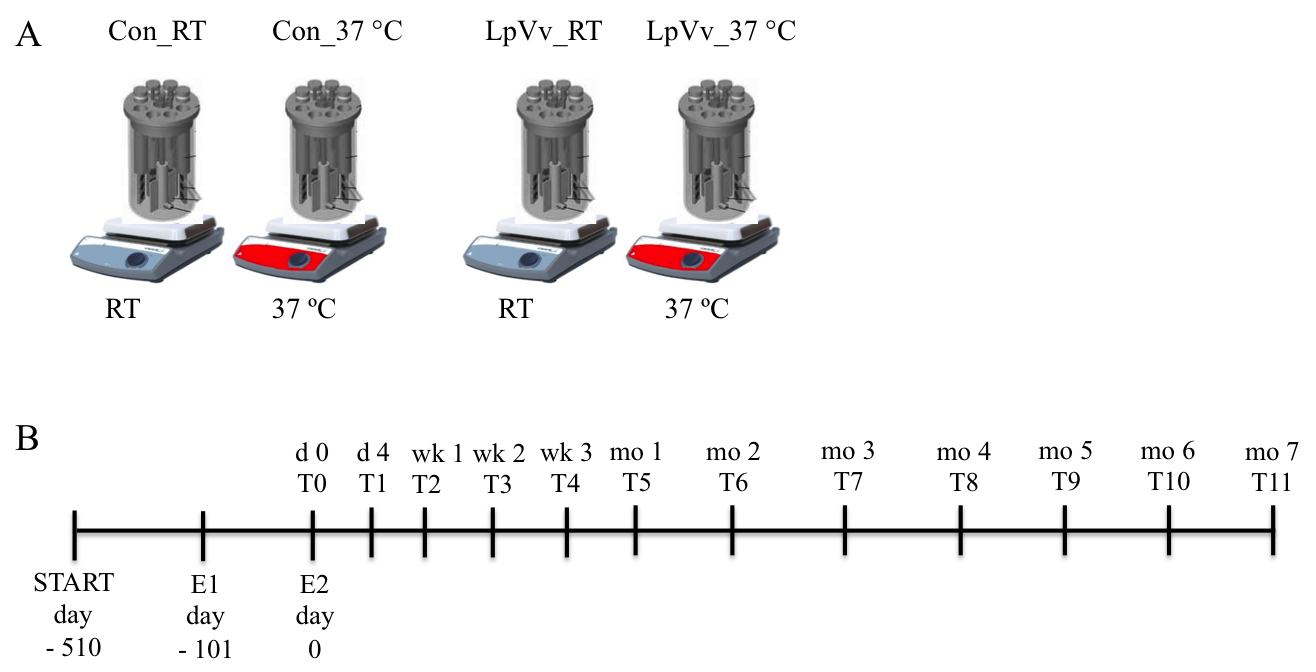

Supplement: Supplementary file 1 — Fig. S1. Biofilm reactor set‐up and experimental timeline. Four CDC biofilm reactors were operated as described in Materials and Methods (A). Drinking water biofilms were allowed to develop on the copper (Cu) surfaces within all four reactors (START) for 510 days under ambient/room temperature (RT) conditions (B) before any manipulation of the reactors occurred. At event 1 (E1), temperatures within two of the reactors, that would serve as the control, mock‐inoculated (Con) and L. pneumophila and V. vermiformis (LpVv)‐inoculated reactors, were elevated to 37°C for the duration of the experiment (Con_37°C and LpVv_37°C, respectively). Biofilms were allowed 101 days to adjust to the temperature change before any further manipulation of the reactors occurred. Prior to inoculation, biofilm material was collected from duplicate Cu coupons from each reactor at T0. After T0 sampling, event 2 (E2) consisted of inoculating 48 h co‐cultures of Lp and Vv into reactors incubated at RT and 37°C, LpVv_RT and LpVv_37°C, respectively. Control reactors, incubated at RT and 37°C, were inoculated with sterile, co‐culture buffer (Con_RT and Con_37°C respectively). Biofilm material was collected from duplicate Cu coupons from each reactor at the time points indicated (B). [file MBT2-10-773-s001.tif]

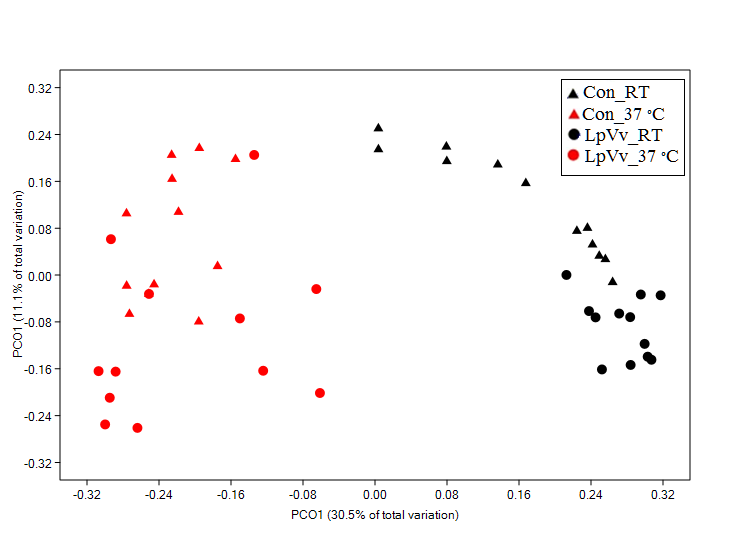

Supplement: Supplementary file 2 — Fig. S2. Principal Coordinate Analysis (PCoA) for sequences derived from biofilm samples. The PCoA graph indicates the contribution of temperature (x‐axis) and effect of inoculation (Con versus LpVv, y‐axis) on the overall variation of the biofilm reactor sequence libraries at each of the twelve time points. [file MBT2-10-773-s002.tif]
