# Supplementary material for: Effect of temperature and colonization of Legionella pneumophila and Vermamoeba vermiformis on bacterial community composition of copper drinking water biofilms
Source: Microb Biotechnol. 2017 Jan 18;10(4):773–88. doi: 10.1111/1751-7915.12457 (PMC5481522; doi:10.1111/1751-7915.12457)
Supplement: Supplementary file 3 — Appendix S1. Diversity index formula and descriptions. [file MBT2-10-773-s003.docx]

**Supplemental Information**

Shannon:


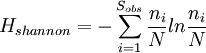


where,


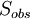
= the number of observed OTUs


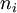
= the number of individuals in OTU *i*


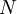
= the total number of individuals in the community

Phylogenetic Diversity (PD)

The phylo.diversity command (implemented in MOTHUR) calculates alpha diversity as the total of the unique branch length.
